# Preparedness needs research: How fundamental science and international collaboration accelerated the response to COVID-19

**DOI:** 10.1371/journal.ppat.1008902

**Published:** 2020-10-09

**Authors:** Cormac M. Kinsella, Pauline Dianne Santos, Ignacio Postigo-Hidalgo, Alba Folgueiras-González, Tim Casper Passchier, Kevin P. Szillat, Joyce Odeke Akello, Beatriz Álvarez-Rodríguez, Joan Martí-Carreras

**Affiliations:** 1 Laboratory of Experimental Virology, Department of Medical Microbiology and Infection Prevention, Amsterdam UMC, University of Amsterdam, Amsterdam, the Netherlands; 2 Institute of Diagnostic Virology, Friedrich-Loeffler-Institut, Greifswald-Insel Riems, Germany; 3 Charité –Universitätsmedizin Berlin, Corporate Member of Freie Universität Berlin, Humboldt-Universität zu Berlin, and Berlin Institute of Health, Institute of Virology, Berlin, Germany; 4 Department of Discovery and Technology, MSD Animal Health, Boxmeer, the Netherlands; 5 School of Molecular and Cellular Biology, Faculty of Biological Sciences, University of Leeds, Leeds, United Kingdom; 6 Institute for Infectious Diseases, University of Bern, Bern, Switzerland; 7 Biology Division, Spiez Laboratory, Swiss Federal Office for Civil Protection, Spiez, Switzerland; 8 Graduate School for Cellular and Biomedical Sciences, University of Bern, Bern, Switzerland; 9 Laboratory of Clinical and Epidemiological Virology, Department of Microbiology, Immunology and Transplantation, Rega Institute for Medical Research, KU Leuven, Leuven, Belgium; University of Pittsburgh, UNITED STATES

## Abstract

The first cluster of patients suffering from coronavirus disease 2019 (COVID-19) was identified on December 21, 2019, and as of July 29, 2020, severe acute respiratory syndrome coronavirus 2 (SARS-CoV-2) infections have been linked with 664,333 deaths and number at least 16,932,996 worldwide. Unprecedented in global societal impact, the COVID-19 pandemic has tested local, national, and international preparedness for viral outbreaks to the limits. Just as it will be vital to identify missed opportunities and improve contingency planning for future outbreaks, we must also highlight key successes and build on them. Concomitant to the emergence of a novel viral disease, there is a ‘research and development gap’ that poses a threat to the overall pace and quality of outbreak response during its most crucial early phase. Here, we outline key components of an adequate research response to novel viral outbreaks using the example of SARS-CoV-2. We highlight the exceptional recent progress made in fundamental science, resulting in the fastest scientific response to a major infectious disease outbreak or pandemic. We underline the vital role of the international research community, from the implementation of diagnostics and contact tracing procedures to the collective search for vaccines and antiviral therapies, sustained by unique information sharing efforts.

## Introduction

Since 1950, the global population has tripled to 7.8 billion, with expansion in meat consumption and living area thought to increase human exposure to microbes infecting wildlife, with occasional ‘spillover’ to people [1]. Approximately 60% of human infectious diseases are zoonotic [2] (transmitted from animals). Zoonoses capable of human-to-human transmission can emerge as catastrophic diseases; acquired immunodeficiency syndrome (AIDS) alone has killed an estimated 32 million [3], whilst coronavirus disease 2019 (COVID-19) has caused the largest global economic crisis since the Great Depression [4].

Speed of response is critical with respect to mitigation of infectious outbreaks, since in a susceptible population, the number of infections may increase exponentially. The aim of outbreak preparedness is therefore to ensure that contingency planning is in place beforehand to avoid delays associated with an ad hoc response, thus enabling intervention whilst case numbers are low. For well-established diseases such as measles in humans, this preparation includes systems for (1) **detection** by routine surveillance, (2) **confirmation** by designated specialist laboratories, and (3) **response**, which will be uniquely tailored to the situation [5]. Contrastingly, spillover of a novel pathogen entails additional complications caused by gaps in fundamental knowledge and validated technical resources. Since this ‘research and development gap’ impinges on the effectiveness and rate of response, it is an important factor that must be considered in the context of preparedness [6].

## The novel pathogen research and development landscape

**Fig 1:**
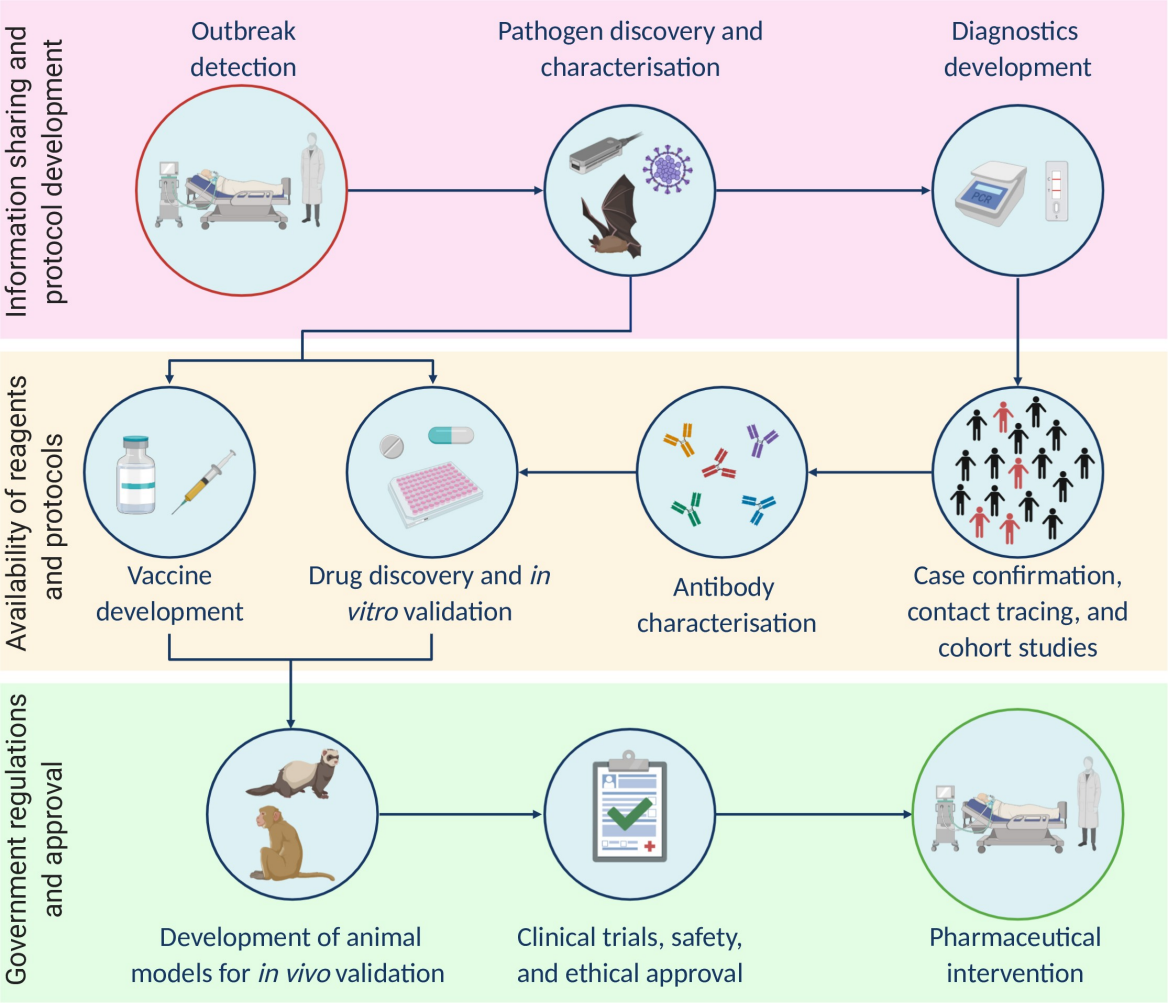
Key components of the research response to viral outbreaks are shown as a simplified workflow. Factors complicating the delivery of each step are annotated on the left.

An outbreak caused by a novel pathogen requires a rapid research effort across the experimental and clinical spectrum. As an outbreak response progresses, research and development continue to play an integral role at each stage (summarised in Fig 1). Here, we describe the various aspects of this research and development landscape using COVID-19 as an example, with emphasis on recent factors that have accelerated the response to the outbreak.

## The detection of COVID-19 and the discovery of SARS-CoV-2

When unusually high incidence of a clinical syndrome is observed in a group of patients (a cluster), there may be suspicion of a shared infection, which could constitute an outbreak. Initial investigation must therefore identify what pathogen is present in order to confirm an outbreak and kick-start a response. The first cluster of patients with COVID-19 was identified on December 21, 2019, after which investigations were carried out by the Chinese Center for Disease Control and Prevention and the National Institute of Viral Disease Control and Prevention [7,8]. A diagnostic panel covering 22 human pathogens was run on these patients, with universally negative results [8]. This immediately raised suspicion of a novel pathogen, requiring discovery methods.

**Fig 2:**
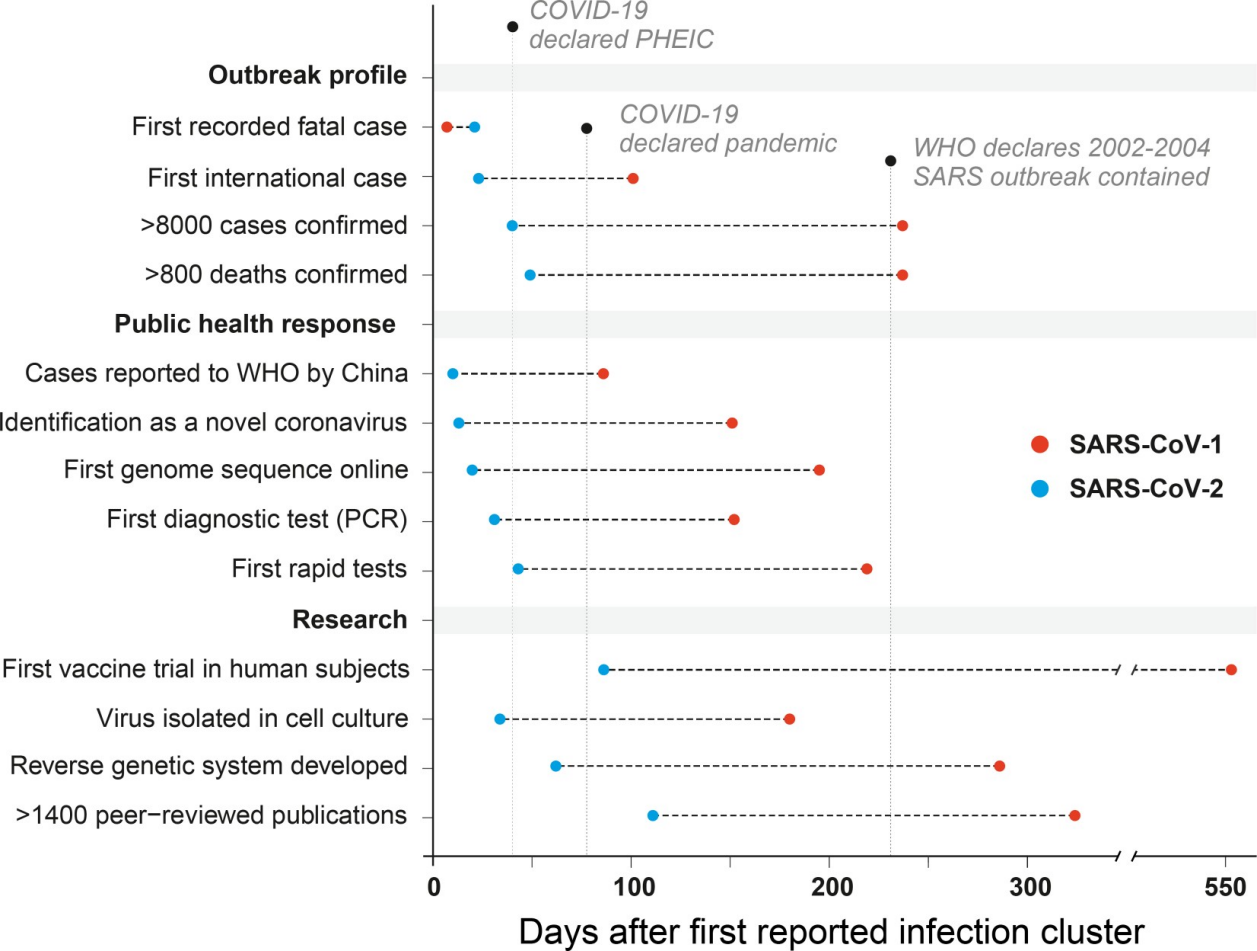
The changing dynamics of research response: contrasting the SARS-CoV-1 and SARS-CoV-2 outbreaks. COVID-19, coronavirus disease 2019; PCR, polymerase chain reaction; PHEIC, Public Health Emergency of International Concern; SARS-CoV-1, severe acute respiratory syndrome coronavirus; SARS-CoV-2, severe acute respiratory syndrome coronavirus 2; WHO, World Health Organization.

Virus discovery methods include polymerase chain reaction (PCR) assays targeting conserved genome regions specific to all members of a taxonomic family (potentially even unknown ones), attempted virus isolation in cell culture, microscopy to study the virion morphology, and metagenomic analysis of human samples for the identification of the pathogen. These analyses were carried out on samples from the initial COVID-19 cluster and published in a remarkably short 34 days [8] (Fig 2). Virus was grown in primary human airway epithelial cell cultures, which provide optimal substrates for coronavirus replication [9,10], and transmission electron microscopy images were generated, showing the spherical virion and surface spikes characteristic of coronaviruses [8]. The first severe acute respiratory syndrome coronavirus 2 (SARS-CoV-2) genomes were published online on January 10, 2020 (Fig 2), 20 days after cluster detection (GISAID accessions EPI_ISL_402119 and EPI_ISL_402121), and phylogenetic analyses showed the virus was a relative of both severe acute respiratory syndrome coronavirus (SARS-CoV-1) [7] (i.e., the etiological agent of the 2002–2004 SARS outbreak) and a strain sampled from a bat [11], confirming a novel zoonotic virus outbreak and kick-starting research efforts worldwide.

## Testing, tracing, and isolating

When facing a novel pathogen, an immediate research priority is the development of diagnostic assays to detect infected individuals. Testing in the clinic ensures that COVID-19 patients are separated from SARS-CoV-2 negative individuals and confirms viral shedding has ceased before discharge. Testing in the community uncovers viral transmission dynamics to support control policies, such as prevalence in vulnerable demographics [12], case fatality [13], or the viral basic reproduction number [14] (i.e., *R*_0_, the average number of further infections arising from 1 case in a naïve population). The first specific PCR assay for SARS-CoV-2 RNA was designed, validated, and published [15] within 13 days of the first genome being made available online (Fig 2). Loop-mediated isothermal amplification (LAMP) assays, which can rapidly and sensitively detect viral RNA or cDNA with minimal equipment, were also validated on patient samples only 2 months after the outbreak emerged [16–18].

Tracing viral spread by identifying infected individuals and their potential contacts enables interventions such as selective quarantine, a method that has long been deployed as a firebreak to epidemic spread. Contact tracing is being applied during the current pandemic; however, with the surge in COVID-19 cases, it has become increasingly challenging to conduct [19]. Technological solutions in the form of privacy-protecting open-source contact tracing apps have emerged rapidly, such as the TraceTogether app of Singapore, released in March 2020. This uses Bluetooth to log other app users that come in close proximity, storing their phone numbers in encrypted form for 14 days, data which are only accessible by the Health Ministry after approach by a contact tracer. With Apple and Google engaged in a similar effort [20], in the future, such software solutions may be integrated with standard phone operating systems and become status quo tools during epidemics.

In addition to diagnostic testing and contact tracing, genomic epidemiology can today be used to describe the evolution of a virus in time and space, uncovering transmission patterns [21]. Importantly, this is now a real-time tool rather than a retrospective one. Whereas the first SARS-CoV-1 genome was published over 6 months into the 2002–2004 outbreak [22] (Fig 2), nearly 5 months into the SARS-CoV-2 pandemic, over 27,000 complete genomes could be downloaded from the GISAID sequence repository (www.gisaid.org). Technological advances in high-throughput sequencing have made this possible, first via short-read technologies such as Solexa (now Illumina), first available in 2006, and second by the 2014 commercial implementation of nanopore sequencing by Oxford Nanopore Technologies. These platforms are commonly used to investigate SARS-CoV-2 transmission clusters, for example, by the COVID-19 Genomics UK (COG-UK) consortium [23], which has sequenced thousands of virus genomes during the pandemic. Due to a relatively low entry price, nanopore technology has been decisive in improving accessibility to sequencing, reinforced by initiatives including the ARTIC Network [24], who quickly developed and published primer schemes and protocols for amplicon sequencing of whole SARS-CoV-2 genomes. Analysis of this wealth of data has also been made more accessible by online tools; for example, the Nextstrain platform [25] is being used to integrate genomic, geographical, and temporal data to visualise patterns of SARS-CoV-2 spread, whilst CoV-GLUE, an online resource created using the Genes Linked by Underlying Evolution (GLUE) [26] software environment, focuses on identifying and tracking new variants.

## Utilising the genome: Fundamental research and development

A viral genome sequence is a launchpad for a range of parallel research goals aiming to deliver technical resources and fundamental knowledge about a new pathogen. The genome provides the sequences of protein-coding genes, which are used to safely produce pure stocks of individual viral proteins. The gene sequence is first amplified from a clinical sample or viral culture using PCR or is synthetically constructed. The DNA is delivered to cells, which produce (express) the protein encoded by the gene. Viral proteins are used in the generation of traditional antibody-based diagnostic assays, such as enzyme-linked immunosorbent assays (ELISAs), which are designed to detect viral proteins in patient samples or patient antibodies against the virus [27]. Although PCR is considered the gold standard for laboratory diagnosis of an active SARS-CoV-2 infection [28], serological assays can detect historical infections and play a key role in determining population attack rates and potentially protective immunity levels [29,30]. Lateral flow assays (LFAs) are used as rapid tests for prior viral exposure and were first reported 2 months after cluster identification [31–33].

Whilst the amino acid sequence of a protein can be accurately predicted from the nucleotide sequence of its encoding gene, the protein conformational structure that determines its function cannot, and must be solved using structural biology techniques. Hardware and data processing performance revolutions in recent years have dramatically accelerated the generation of high-resolution structures; only 2 months after the first SARS-CoV-2 genome was released, a 3.5 Å resolution cryogenic electron microscopy (cryo-EM) structure of the spike protein was published [34], revealing differences to SARS-CoV-1 that preclude the binding of some anti-SARS-CoV-1 antibodies. This was followed in short order by structures including the cellular receptor ACE2 [35] bound to the spike protein [36], the SARS-CoV-2 nucleocapsid RNA-binding domain [37], and the SARS-CoV-2 main protease [38]. This structural information is useful for in silico modelling of protein–protein interactions and high-throughput computational screening of chemical compounds with potential antiviral properties. Another remarkable approach to find drug targets exploited expression of SARS-CoV-2 proteins in human cells, followed by separation of the viral proteins (and bound host proteins) from the host background. Identification of bound host proteins by mass spectrometry revealed a number of druggable candidates that may represent therapeutic targets [39].

In vivo studies such as those in livestock, frugivorous bats, and companion animals are necessary to establish their potential role in forming viral reservoirs and to provide data that are informative on the possible pathways of SARS-CoV-2 transmission from its wild reservoir to humans [40,41]. Furthermore, nonhuman primate models will be key to expediting preclinical evaluation of vaccines and other therapeutics for use in humans [42,43]. Establishment of viral cultures is vital for numerous downstream applications, including generating these in vivo infection models for therapeutic testing [44], antiviral screening [45], and fundamental virus characterisation [46]. Traditionally, a clinical sample containing replication competent virus would be required; however, access to samples for researchers in countries without active cases is often logistically difficult in the early phases of an outbreak. Today, only a genome sequence is required—synthetic DNA technology allows the reconstruction of viral genomes in artificial vectors—from which viable infectious RNA can be produced and replicating virus rescued. The first such SARS-CoV-2 culture was achieved only 1 week after delivery of synthetic DNA constructs spanning the genome [47] and reported 42 days after the first genome became available. This technology offers a powerful platform to carry out reverse genetic approaches using genetic engineering to study genotype-to-phenotype relationships and the function of viral components, and also enables validation of diagnostic tests in the absence of clinical specimens [15].

## Delivering pharmaceutical interventions to the clinic

The future trajectory of the SARS-CoV-2 pandemic is uncertain; however, elimination of the virus may depend on the availability of antiviral treatments and vaccines. Delivery of novel pharmaceutical interventions from conception to clinic is a prolonged process with numerous practical and regulatory hurdles to cross, necessitating short-term stopgaps. Repurposing of drugs already approved for human usage is an attractive option: their production is already optimised and upscaled, and they have known safety and bioavailability data, allowing human clinical trials to proceed rapidly after testing of in vitro efficacy. However, the repurposing of approved drugs can have major consequences in outbreak situations, causing acute shortages and rendering them unavailable to patients using them for the licensed indication [48]. Furthermore, medications can have potentially serious side effects, and emergency use approval may entail an uncertain risk/benefit profile, as seen with the example of hydroxychloroquine, which recently had its emergency use authorisation (EUA) revoked by the United States Food and Drug Administration (FDA) [49].

Clinical trials of therapeutic drugs involving hundreds of COVID-19 patients were ongoing by early February [50]. During March and April, numerous compounds were identified that either have direct action on SARS-CoV-2 in vitro [45,51–53] or may have indirect action via host proteins [39,54], paving the way for in vivo efficacy trials (Fig 1). Furthermore, on the basis of 2 clinical trials, the FDA granted an EUA of remdesivir for COVID-19 treatment on May 1, 2020 [55]. This was the first direct antiviral approval, just over 4 months into the outbreak.

Delivery of neutralising antibodies (nAbs) to patients early in disease is another treatment option. Serum from convalescent COVID-19 patients contains anti-SARS-CoV-2 antibodies, and donated serum can be infused to acutely ill patients [56,57]. Other options include products containing purified polyclonal antibodies from the pooled sera of COVID-19 survivors [58], through to the identification and characterisation of monoclonal nAbs, since these have the potential to be produced in large quantities and delivered as immunotherapy. By February, a SARS-CoV-1 nAb (CR3022) was reported to bind the receptor-binding domain of SARS-CoV-2 spike protein [59]; however, it was later shown not to cross-neutralise the virus [60]. In early April, an antibody (S309) from a SARS-CoV-1 survivor was found to neutralise SARS-CoV-2 [61], followed in May by a humanised antibody (47D11) capable of blocking infection with both SARS-CoV-1 and SARS-CoV-2 in vitro [62]. Also in May, a team demonstrated in vivo protection against high-dose SARS-CoV-2 challenge in Syrian hamsters after injection of a human monoclonal nAb [63], whilst another reported identification of 19 SARS-CoV-2 nAbs [64]. This was accomplished by isolating individual B cells reacting to native-like SARS-CoV-2 spike protein, followed by expression of the encoded antibodies and virus neutralisation assays. Importantly, highly potent nAbs were found that targeted different regions of the spike protein, implying suitability as a cocktail therapy with reduced risk of viral escape.

Vaccines are used to safely increase immunity levels against pathogens in populations in order to protect individuals and reduce pathways to viral spread. For SARS-CoV-2, a remarkably fast and broad vaccine development response was initiated, with the first candidate reaching human trials only 86 days after cluster identification [65], over 6 times faster than the first SARS-CoV-1 human vaccine trial [66] (Fig 2). At the time of writing, 35 candidates had reached human trials, with 122 more in preclinical development [67]. A range of vaccine platforms (different core approaches to elicit antibody-based immunity and their associated manufacturing pipelines) are being trialled [68]. Amongst them are next-generation techniques like mRNA vaccination [65], which requires relatively little development time after viral genome sequencing. If successfully tested and deployed, these novel concepts may establish themselves more firmly within the vaccine ecosystem and potentially accelerate vaccine development beyond the SARS-CoV-2 pandemic. However, the implementation of novel pharmaceutical interventions will ultimately be subject to the normal technical challenges involved with upscaling production to meet massive demand and will be dependent on the availability of appropriate production lines [68]. Many of the novel vaccine approaches currently being trialled have never been manufactured at this scale, suggesting unforeseen scalability issues may place future roadblocks.

## Information, property, and knowledge sharing

Against the backdrop of technological advancements that have boosted outbreak research capacity in recent years, key paradigm shifts in information sharing have also occurred, dramatically increasing how quickly and widely new results and data are received by other researchers and the public. In a rapidly evolving situation, maximum data utility is achieved by prompt release. The pace of viral genome publication during crises was highlighted as a problem during the 2014–2016 Ebola outbreak [69], yet arguably a sea change has occurred since. Furthermore, databases have emerged that compile COVID-19 publications and data resources [70] or provide daily updates on outbreak data worldwide [71–74], accessible to all. For researchers, the open access *bioRxiv* and *medRxiv* preprint servers (launched in 2013 and 2019, respectively) have become invaluable resources for the rapid dissemination of results, hosting a combined 7,060 articles by July 29, 2020. The cost of this speed is the absence of peer review, which can sometimes result in the publication of data falling below a necessary quality standard. Encouragingly, this openness has not been limited to academic spheres; many major technology companies including Intel, Microsoft, and Amazon have temporarily granted open access to their patent libraries for SARS-CoV-2-related research as part of the ‘Open COVID Pledge’ [75] to encourage innovation via sharing of intellectual property. Some pharmaceutical companies have also made similar moves, from AbbVie (North Chicago, USA) agreeing not to enforce patent rights on a drug in COVID-19 trials [76] to Roche (Basel, Switzerland) sharing the composition of a buffer in their diagnostic kit with the Dutch government [77]. Numerous community-driven efforts have also sprouted, from 3D printing and donation of personal protective equipment [78] to the repurposing of company production lines to manufacture and donate essential materials such as hand sanitiser [79].

Timely and high-quality scientific research relies on knowledge sharing between teams with varying expertise and facilities, making connectivity a keystone component of research preparedness for outbreaks. Whilst the World Health Organization (WHO) plays a crucial role in coordinating global research efforts in response to the COVID-19 pandemic, preexisting networks and formal consortia of trusted partners can immediately coordinate to form focus groups and share resources [15]; several of these have been formed in response to major epidemics of recent years (Table 1). Some of these, such as Platform for European Preparedness Against (Re-)emerging Epidemics (PREPARE) [80] and HONOURs [81], specifically train research preparedness concepts to the next generation of scientists. An advantage of larger networks is the ability to coordinate multi-armed studies with unified procedures and centralised administration, in order to maximise sample size and achieve consensus faster. For this purpose, WHO launched the Solidarity clinical trial for testing COVID-19 treatments [82], which involves over 100 countries and intends to complete 80% faster than a typical randomised clinical trial. Representative national ethics committees supported by WHO, funding agencies, and relevant collaborative research consortia provided statements guiding the conduct of COVID-19 research, which helps to standardise and expedite ethical research [83]. Another important contribution of international collaborations, such as the COVID-19 Clinical Research Coalition (CRC) (Table 1), is the acceleration of COVID-19 research in low-to-middle-income countries (LMIC) where research resources, laboratory facilities, and manufacturing infrastructures are commonly limited relative to high-income countries [84].

**Table 1:** A selection of the many networks and consortia conducting research into SARS-CoV-2 and COVID-19.

Networks	Focus	Website
ACTIV	Accelerating COVID-19 Therapeutic Interventions and Vaccines	https://www.nih.gov/research-training/medical-research-initiatives/activ
ARTIC Network	Real-time molecular epidemiology for outbreak response	https://artic.network/
CEPI	Coalition for Epidemic Preparedness Innovations	https://cepi.net/
COMPARE	COllaborative Management Platform for detection and Analyses of (Re-)emerging and foodborne outbreaks in Europe	https://www.compare-europe.eu/
COPCOV	Chloroquine/ hydroxychloroquine prevention of Coronavirus Disease (COVID-19) in the healthcare setting; a randomised, placebo-controlled prophylaxis	https://www.tropmedres.ac/covid-19/copcov
COV-IRT	COVID-19 International Research Team	https://covirt19.org/
COVID-19 CRC	COVID-19 Clinical Research Coalition	https://covid19crc.org/
ECRAID	European Clinical Research Alliance on Infectious Diseases	https://www.ecraid.eu/
eMERGE	Electronic Medical Records and Genomics	https://emerge-network.org/
GLOPID-R	Global Research Collaboration for Infectious Disease Preparedness	https://www.glopid-r.org/
HONOURs	Host switching pathogens, infectious outbreaks, and zoonosis	https://www.honours.eu/
IDCRC	Infectious Diseases Clinical Research Consortium	https://med.emory.edu/departments/medicine/divisions/infectious-diseases/idcrc/index.htm
INITIATE	Innate-Immunometablism as Antiviral Target	https://initiate-itn.eu/
ISARIC	International Severe Acute Respiratory and emerging Infection Consortium	https://isaric.tghn.org/
PANDORA	Pan-African Network For Rapid Research, Response and Preparedness for Infectious Diseases Epidemics	https://pandora.tghn.org/
PREPARE	Platform for European Preparedness Against (Re-)emerging Epidemics	https://www.prepare-europe.eu/
RECOVER	Understand the COVID-19 pandemic through clinical research in order to transform patient care and public health responses	https://www.recover-europe.eu/
Solidarity	International clinical trial to help find an effective treatment for COVID-19, launched by WHO and partners	https://www.who.int/emergencies/diseases/novel-coronavirus-2019/global-research-on-novel-coronavirus-2019-ncov/solidarity-clinical-trial-for-covid-19-treatments
VIRUS-X	Viral metagenomics for innovation value	http://virus-x.eu/
ZAPI	Zoonotic Anticipation and Preparedness Initiative	https://www.imi.europa.eu/projects-results/project-factsheets/zapi

ACTIV, Accelerating COVID-19 Therapeutic Interventions and Vaccines; CEPI, Coalition for Epidemic Preparedness Innovations; COMPARE, COllaborative Management Platform for detection and Analyses of (Re-)emerging and foodborne outbreaks in Europe; COPCOV, Chloroquine/ hydroxychloroquine prevention of Coronavirus Disease (COVID-19) in the healthcare setting; COVID-19, coronavirus disease 2019; COVID-19 CRC, COVID-19 Clinical Research Coalition; COV-IRT, COVID-19 International Research Team; ECRAID, European Clinical Research Alliance on Infectious Diseases; eMERGE, Electronic Medical Records and Genomics; GLOPID-R, Global Research Collaboration for Infectious Disease Preparedness; IDCRC, Infectious Diseases Clinical Research Consortium; INITIATE, Innate-Immunometablism as Antiviral Target; ISARIC, International Severe Acute Respiratory and emerging Infection Consortium; PANDORA, Pan-African Network For Rapid Research, Response and Preparedness for Infectious Diseases Epidemics; PREPARE, Platform for European Preparedness Against (Re-)emerging Epidemics; RECOVER, Rapid European COVID-19 Emergency Response research; SARS-CoV-2, severe acute respiratory syndrome coronavirus 2; WHO, World Health Organization; ZAPI, Zoonotic Anticipation and Preparedness Initiative.

## Outlook

The SARS-CoV-2 pandemic has been an unprecedented test of novel pathogen outbreak preparedness, revealing strengths and weaknesses in all domains. As we have highlighted here, research capacity to respond to novel outbreaks is at an all-time high. The massive threat that viral outbreaks pose to lives and economies underscores the need to build on this success by promoting fundamental research capacity and networks of collaboration.
